# Friction injury of the central vein caused by catheter for hemodialysis: an in vitro study

**DOI:** 10.1038/s41598-024-56485-5

**Published:** 2024-03-10

**Authors:** Zhaoxing Wang, Kunpeng Wang, Yan Xu

**Affiliations:** 1 Department of Nephrology, Emergency General Hospital, Beijing, 100028 China; 2 School of Mechatronic Engineering and Automation, Shanghai University, Shanghai, 200444 China

**Keywords:** Hemodialysis, Central venous catheters, Vascular system injuries, Friction, Vascular endothelium, In vitro techniques, Biophysics, Medical research, Nephrology, Materials science

## Abstract

Vascular injury such as central venous stenosis (CVS) is a common complication in hemodialysis patients with central venous catheters (CVCs), yet the impact of the microstructure and partial physic characteristics of catheter surface on the chronic injury of central vein has not been elucidated. In this study, the microscopic morphology of tips and bodies of six different brands of polyurethane CVCs was observed and their roughness was assessed. Subsequently, an in vitro model was established to measure the coefficients of friction (COF) between CVCs (tips and bodies) and the vena cava intima of Japanese rabbits under the same condition in a linear reciprocating mode, and changes in the intima of vessels after friction were observed. The study found that there was a significant variation in surface roughness among different brands of CVCs (tips *P* < 0.001, bodies *P* = 0.02), and the COF was positively correlated with the catheter surface roughness (tips *P* = 0.005, *R* = 0.945, bodies *P* = 0.01, *R* = 0.909). Besides, the endovascular roughness increased after friction. These findings suggest that the high roughness surface of CVCs may cause chronic mechanical friction injury to the central venous intima, which is one of the potential factors leading to CVS or occlusion. This provides a breakthrough for reducing complications, improving patient prognosis, and advancing catheter surface lubrication technology.

## Introduction

In many countries, maintenance hemodialysis (MHD) is the main renal replacement therapy for patients with end-stage kidney disease. Abnormal functioning of hemodialysis (HD) vascular access is one of the important factors that threaten the quality of dialysis and the long-term prognosis of this population^1^. Although the KDOQI Guideline for Vascular Access: 2019 Update still does not consider central venous catheters (CVCs) as the preferred vascular access for HD^2^, the real proportion is much higher, particularly in older patients and patients with diabetes, and > 60% of patients’ first HD access is CVC^3,4^. In addition, > 50% of the patients on home HD have a CVC as their initial vascular access^5,6^. Moreover, recent studies have found that CVCs are not directly associated with mortality^7,8^. In general, many patients on MHD have to select CVCs, whose adverse effects including different vascular injuries are worthy of great concern. Acute mechanical injury of blood vessels may occur during catheterization, and other permanent injuries including central venous stenosis (CVS), mural thrombosis, and vascular perforation were also observed^9,10^. Fasika et al. observed that CVS occurs in approximately 10% (95% CI 8–13%) of patients with chronic kidney disease (CKD) and 13% (95% CI 10–17%) of patients with CKD and tunneled central venous dialysis catheters^11^. In particular, CVS can induce devastating consequences such as venous return blockage and vascular access dysfunction. Unfortunately, its mechanism has confused us. Previous studies have suggested that the catheter or the guiding medium may cause acute mechanical friction damage to the vascular intima during insertion^12,13^. Moreover, factors such as the position or morphology of the catheter tip, the size of the catheter relative to the central vein, and changes in right atrial hemodynamics are also associated with catheter-related complications such as CVS^14–16^. In addition, imaging confirmed that the catheter had sustained regular or irregular multidirectional displacement in the blood vessel because of blood flow, heartbeat, breathing, body position, and regular negative pressure suction produced by the blood pump of the dialysis machine^17,18^. Persistent mechanical friction injuries to the vascular intima caused by moving CVCs may be the one of initiating factors of subsequent venous stenosis^19^. According to previous studies, we hypothesized that sustained mechanical friction damage to the vascular endothelium induced by a CVC for HD initiates a cascade of downstream injuries.

To our knowledge, no study has focused on mechanical friction damage of CVCs for HD to the vascular intima. Meanwhile, the medical device regulatory department has not established reference standards for the surface roughness of catheters, friction coefficient, etc. Therefore, basic and clinical studies relevant to this field are urgently needed. The aims of this study were to ① observe and analyze the microscopic morphology of CVCs for HD with different technologies, ② measure the friction coefficients on different parts of different catheters, ③ design an in vitro model for simulating the friction of CVCs against the vascular intima and then observe morphological changes in the vascular intima before and after the friction to estimate whether friction damage exists, and ④ confirm whether the friction coefficient of different catheters/CVC parts and mechanical friction damage are correlated, which is an entry to revealing whether mechanical friction damage of CVCs for HD to the vascular intima initiates or participates in CVS development. The results of this study may provide theoretical support for improving CVC technology in the future.

## Results

### Micromorphology of catheters

**Figure 1:**
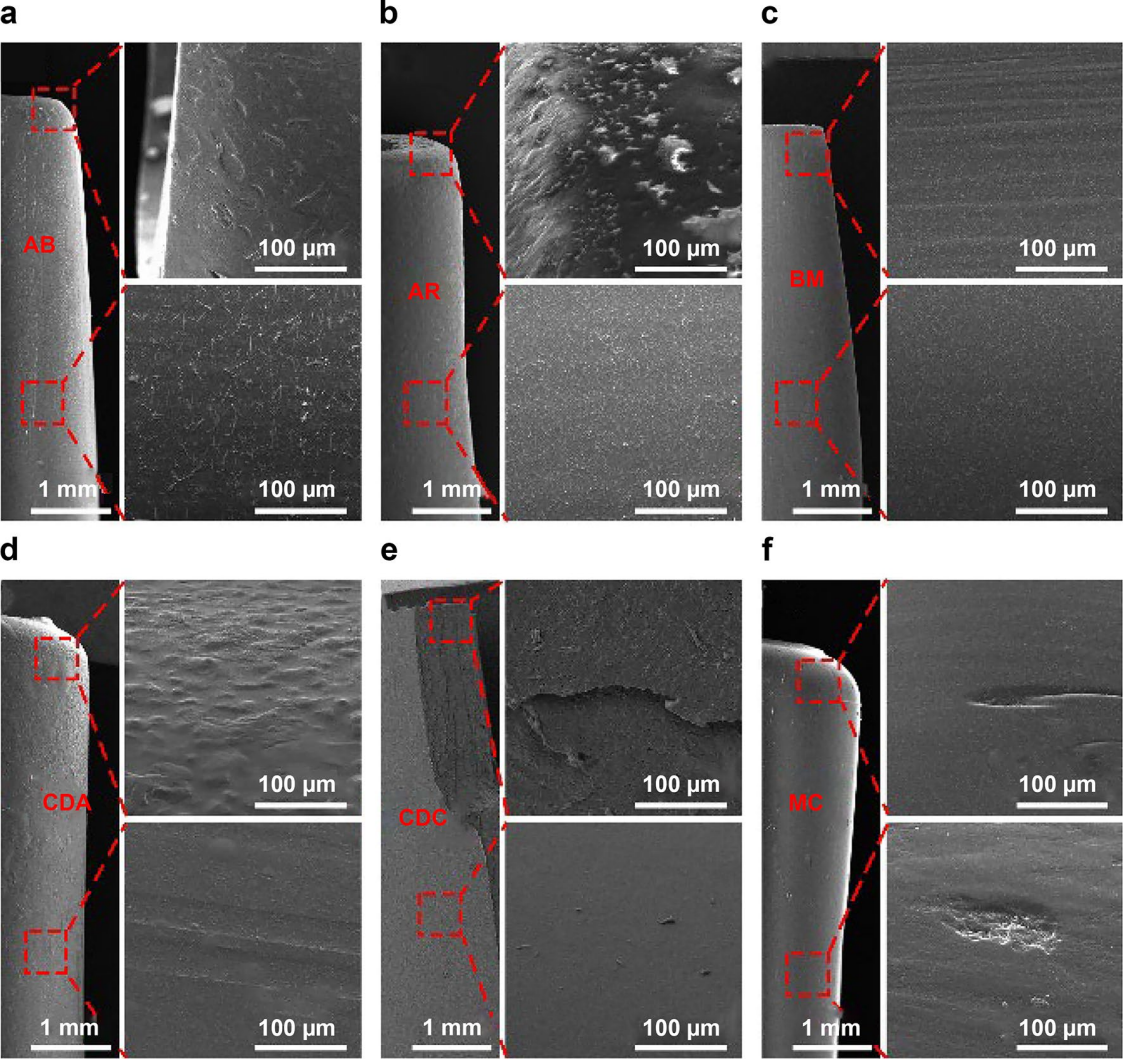
Micromorphology of tips and bodies of six testing catheters (AB, AR, BM, CDA, CDC, and MC). In general, most of bodies were smoother than their tips under SEM.

The surfaces of six experimental catheters were flat and smooth when observed by the naked eye. However, the microstructures of catheter surfaces were completely different when using SEM. In the low magnification image, the surfaces were smooth and delicate, whereas in the high magnification image, the surfaces showed a relatively rough and diverse micromorphology regardless of whether the tips or bodies were smooth (Fig. 1). These morphological manifestations are the physical basis for possible mechanical friction damage to the vascular intima caused by the catheter.

### Surface roughness assessment of catheters

**Figure 2:**
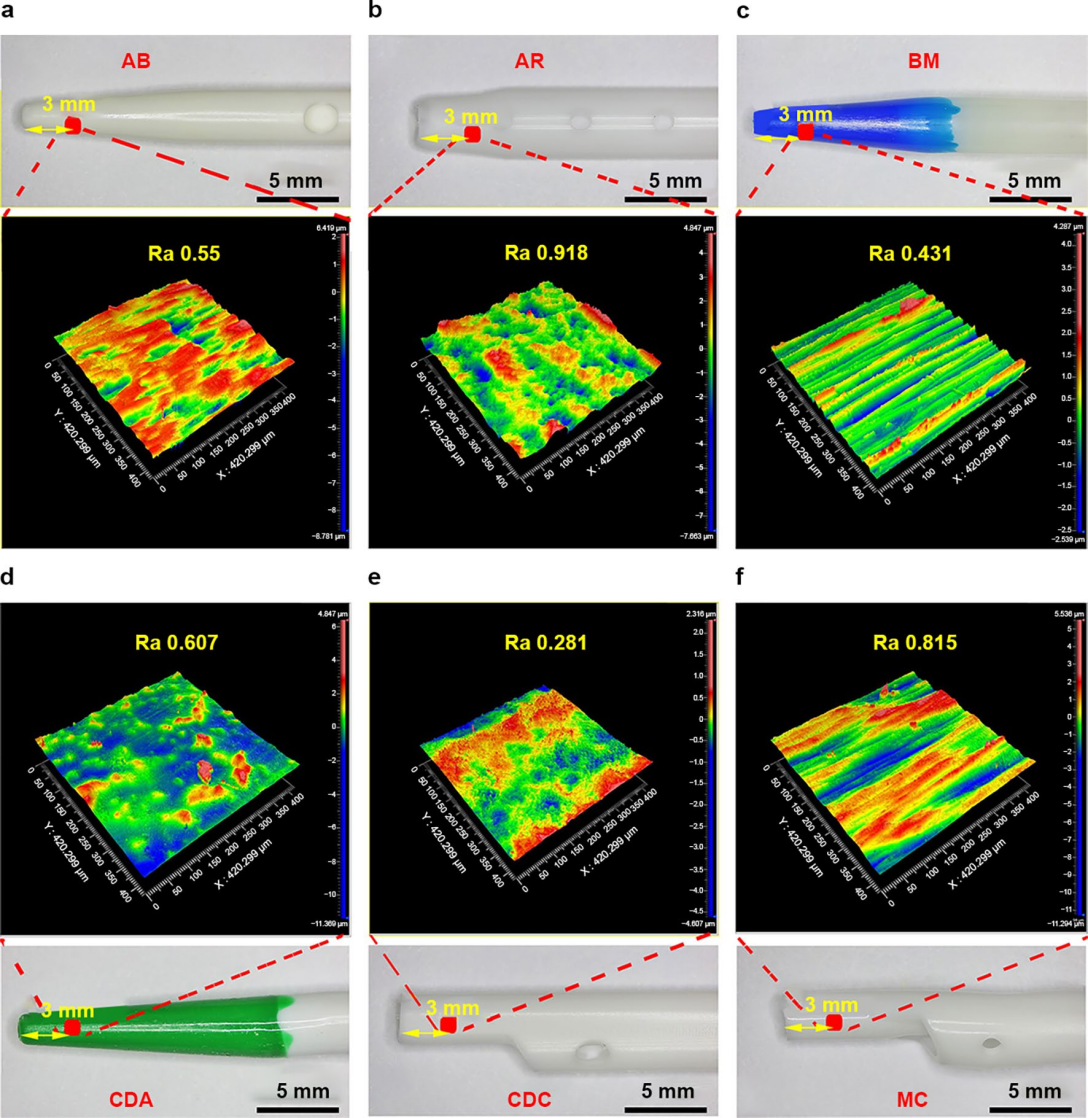
Roughness of different catheter tips and average Ra values of multiple measurements (Overall comparison between groups *P* < 0.001).

**Figure 3:**
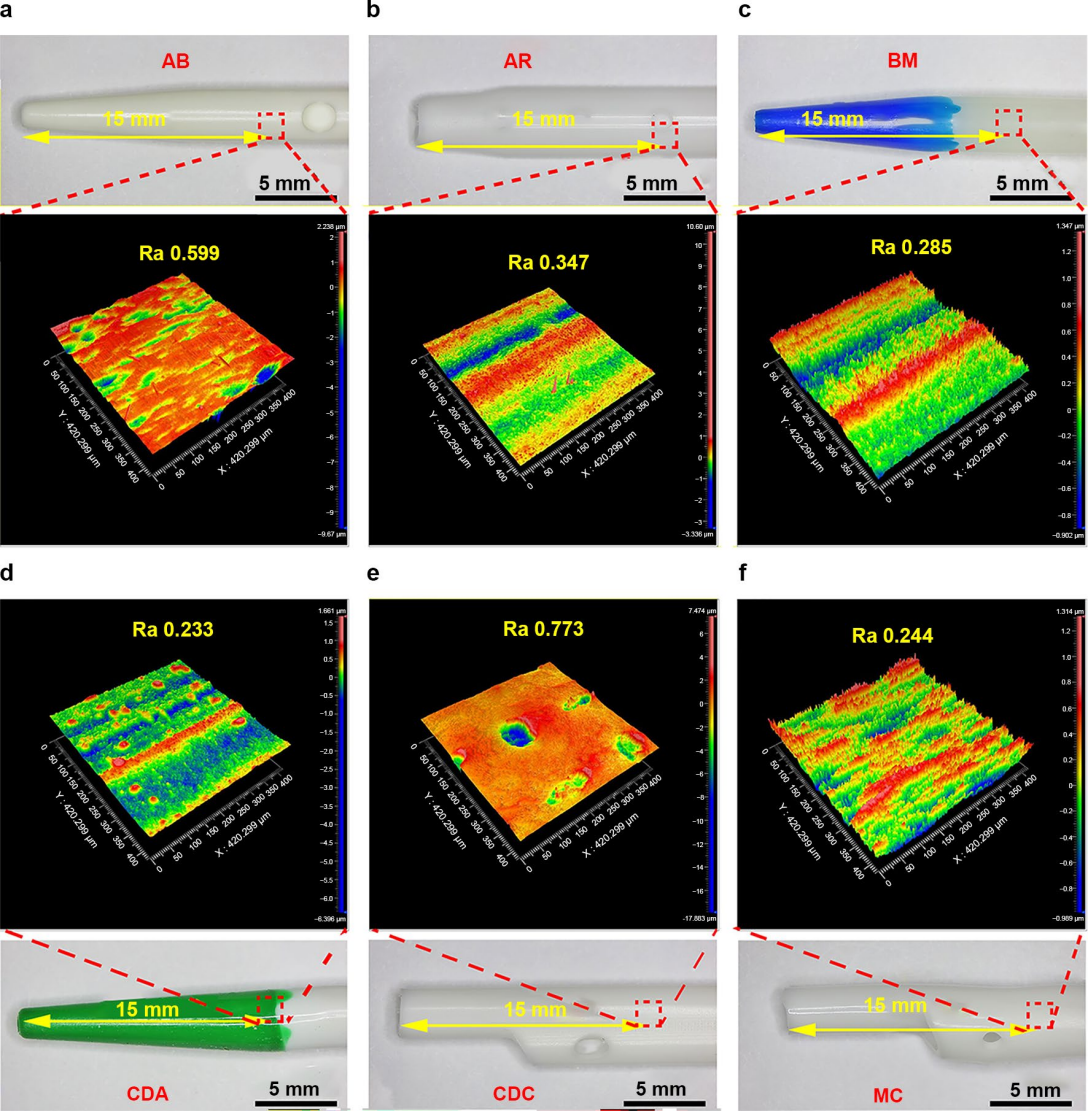
Roughness of different catheter bodies and average Ra values of multiple measurements (Overall comparison between groups *P* = 0.02).

White-light interference technology is an essential and elaborate method to measure the surface roughness of materials. In this study, a 3D white-light interferometry was used to quantitatively evaluate the surface roughness of catheter tips (Fig. 2) and bodies (Fig. 3). To ensure data reliability and accuracy, multiple measurements were taken at different sampling sites. The results illustrated that the mean deviation of the roughness arithmetic (Ra) values of the tip of all experimental catheters ranged from 0.262 to 0.969 μm, whereas it ranged from 0.162 to 1.151 μm in catheter bodies. Higher Ra values indicate rougher surfaces. The result of white-light interferometry showed significant differences in roughness between tips or bodies of catheters from different brands (tips *P* < 0.001, bodies *P* = 0.02), and the roughness of the tip surface of most catheters was significantly higher than that of the body surface (CDA* P* = 0.01, CDC* P* = 0.01, MC* P* = 0.01, AR* P* < 0.001), which may be related to the irregular shape and processing technique of tips.

### Correlation between roughness and coefficients of friction (COF) of catheters

**Figure 4:**
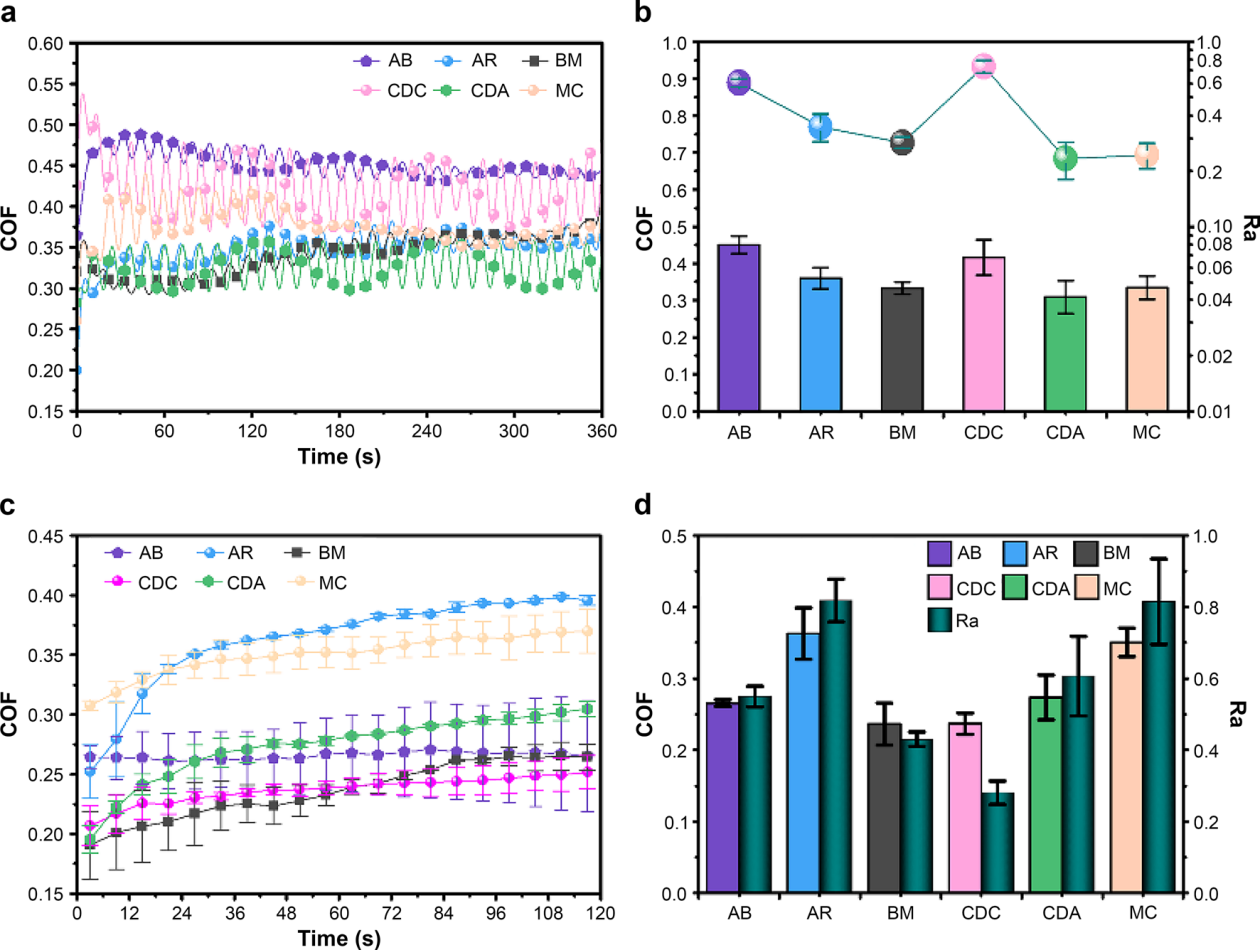
Correlation between the COFof the catheter bodies (**a**) and their roughness (**b**) (*P* = 0.01, *R* = 0.909). Correlation between the COFof the catheter tips (**c**) and their roughness (**d**) (*P* = 0.005, *R* = 0.945).

By using UMT5 and the proposed in vitro model, the COF of bodies (Fig. 4a) and tips (Fig. 4c) of different catheters was measured through linear friction with preset parameters between the catheters and the vascular intima. Then, correlation analyses between the roughness of catheter bodies (Fig. 4b), tips (Fig. 4d) and COF were conducted. The results indicated that the roughness of catheter bodies and tips showed a significant positive correlation with their respective COF. The correlation coefficient (R-value) between Ra and COF of catheter bodies is 0.909 (*P* = 0.01); for Ra and COF of catheter tips, the R-value is 0.945 (*P* = 0.005). That is, under the same contact pressure, the larger the roughness of catheter bodies or tips, the higher COF between catheters and the vascular intima, which confirms that a catheter with a relatively rough surface topography will generate a greater force of friction when displaced relative to the vessel intima.

### Morphological changes of the vascular intima before and after friction induction

After linear friction was conducted between different catheters and vascular intima with UMT5, obvious morphological changes of the vascular intima were observed under SEM. The white-light interferometry was used to assess the vascular intima, it was found that the roughness changed greatly compared with that before friction induction.

**Figure 5:**
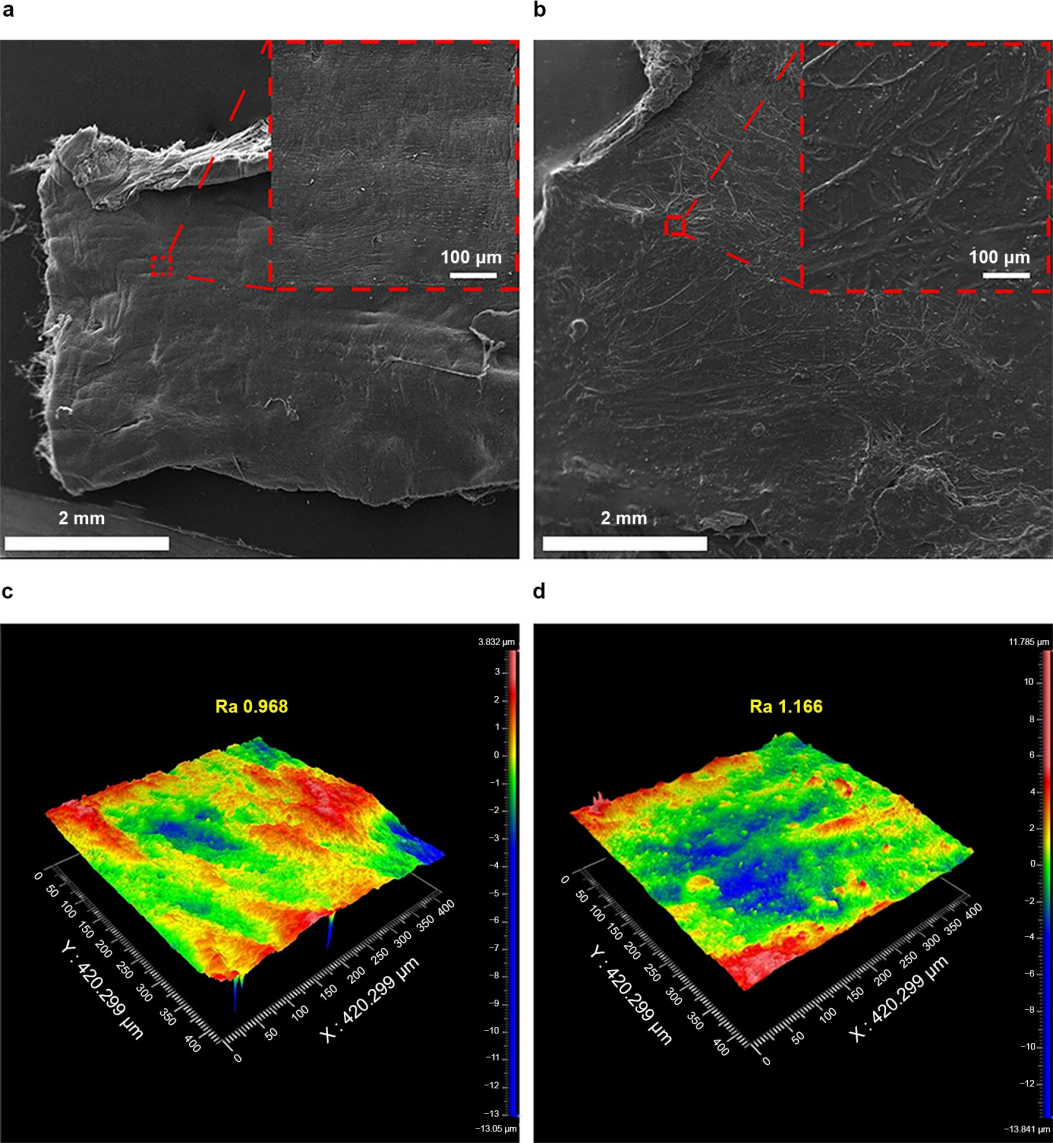
Morphology (**a**) and roughness (**c**) of the vascular intima before friction induction and morphology (**b**) and roughness (**d**) of the vascular intima after friction.

Figure 5 shows the situation of the vascular intima after the friction of the CDA catheter body with the minimum surface roughness. Given the large differences in roughness at different locations of blood vessels after friction and the need for dehydration and freeze-drying of blood vessels before applying SEM, quantitative comparisons of changes in the morphology and roughness of different vessels before and after friction induction were difficult. However, the results of this study can definitely confirm that in these in vitro simulation experiments, all experimental catheters caused mechanical friction damage to the vascular intima.

## Discussion

CVC is the initial or long-term vascular access for most patients with MHD. CVS or occlusion secondary to catheter insertion causes permanent damage to blood vessels and many serious problems related to the obstruction of central venous return. Moreover, it accelerates vascular access resource depletion, seriously affecting the quality of dialysis and long-term prognosis^20^.

Unfortunately, the exact pathogenesis of CVC-related CVS remains undetermined. The possible pathogenic mechanisms proposed so far include mechanical injury during catheter insertion, turbulence of blood flow in the location of the intravascular catheter, coagulation cascade and inflammation caused by prolonged catheter indwelling^19,21^. In addition, Alian et al. suggested that CVS significantly correlated with the retention time of catheters with a Dacron cuff^22^. Forauer et al. conducted a pathological study of the vascular intima of patients with central venous catheterization and found that local damage to the vascular intima, including intimal denudation and adhesion thrombus, was observed within 14 days after catheter insertion. Meanwhile, long-term catheterization (> 90 days) induced thickening of the venous intima, and thrombus, collagen and endothelial cells in different stages can be found in the area where the catheter contacts the venous intima^23^. These phenomena suggest that CVC-induced CVS is a chronic and progressive pathology that cannot be explained simply by puncture injury. Based on the results of studies on urethral friction damage caused by urinary catheters, friction injury caused by cardiovascular catheters on the blood vessel, tests on surface lubrication, characteristics of various movements, and long-term indwelling of CVCs for dialysis in the central vein, CVCs for HD will also cause mechanical friction damage to the inner membrane of the central vein^24–26^. For the first time, Leblanc et al. proposed that the stiffness of the catheter might be relevant to the initiation or progression of the vascular intima wear or catheter-induced irritation, leading to chronic vascular endothelial injury^27^. An earlier study also suggested that relatively soft silicone catheters were associated with lower incidence of thrombosis than stiffer polyethylene dialysis catheters^28^, which also indirectly confirmed that different stiffnesses of catheters had different effects on vascular endothelial injury. Some studies have also reported delayed vascular injury after catheterization, and progressive vascular injury in these cases can be present up to 35 days after catheterization^29^. The above studies have directly or indirectly indicated that CVC indwelling is related to chronic mechanical friction injury of the vascular intima.

When chronic mechanical friction trauma occurs, local inflammatory responses and oxidative stress in damaged blood vessel walls are triggered, resulting in the release of peroxidase and activation of the thrombin cascade, and promoting endothelial hyperplasia, central vein thrombosis, vascular stenosis or occlusion and other serious complications^19^. Studies have revealed that approximately 20 to 40% of patients with CVCs on MHD were diagnosed with CVS^30^. Meanwhile, some patients without any clinical symptoms can only be accidentally found to have CVS related to the location of the original catheter during imaging examination months or years after CVC removal. This indicated that the real incidence of CVC-induced CVS may be higher than the existing estimated data. Lin et al. conducted in vitro tests, mimicking the reciprocating sliding motion between a blood vessel and a catheter, and indicated that decreasing catheter stiffness and endothelial glycocalyx layer (EGL) degradation were the strongest factors that increased the coefficient of friction at the aorta–catheter interface^31^. EGL damage is related to inflammation, oxidative stress, and other vascular damage factors.

Thus, we speculate that CVC indwelling may cause chronic mechanical friction injury to the vascular intima, which may be a crucial initiating or inducing factor for subsequent inflammation and oxidative stress in damaged blood vessels, and central venous thrombosis, stenosis, or occlusion.

To explore the relationship between the surface roughness of the catheter and vascular intima trauma, material surface observation and in vitro friction experiments were conducted. By combining the characteristics of the catheter displacement in vivo observed through catheter angiography and clinical experience, we considered that the friction between the catheter and the vascular intima is mainly linear, the contact loading force between the two surfaces is small, and the friction speed is relatively slow. Owing to the presence of blood in the vein, a mixed friction mode exists between the catheter and the vascular intima. According to our previous experience, we immersed blood vessels in 0.9% saline solution in the in vitro experiments to simulate the frictional environment between the catheter and the inner membrane in vivo as much as possible under CVC indwelling.

**Figure 6:**
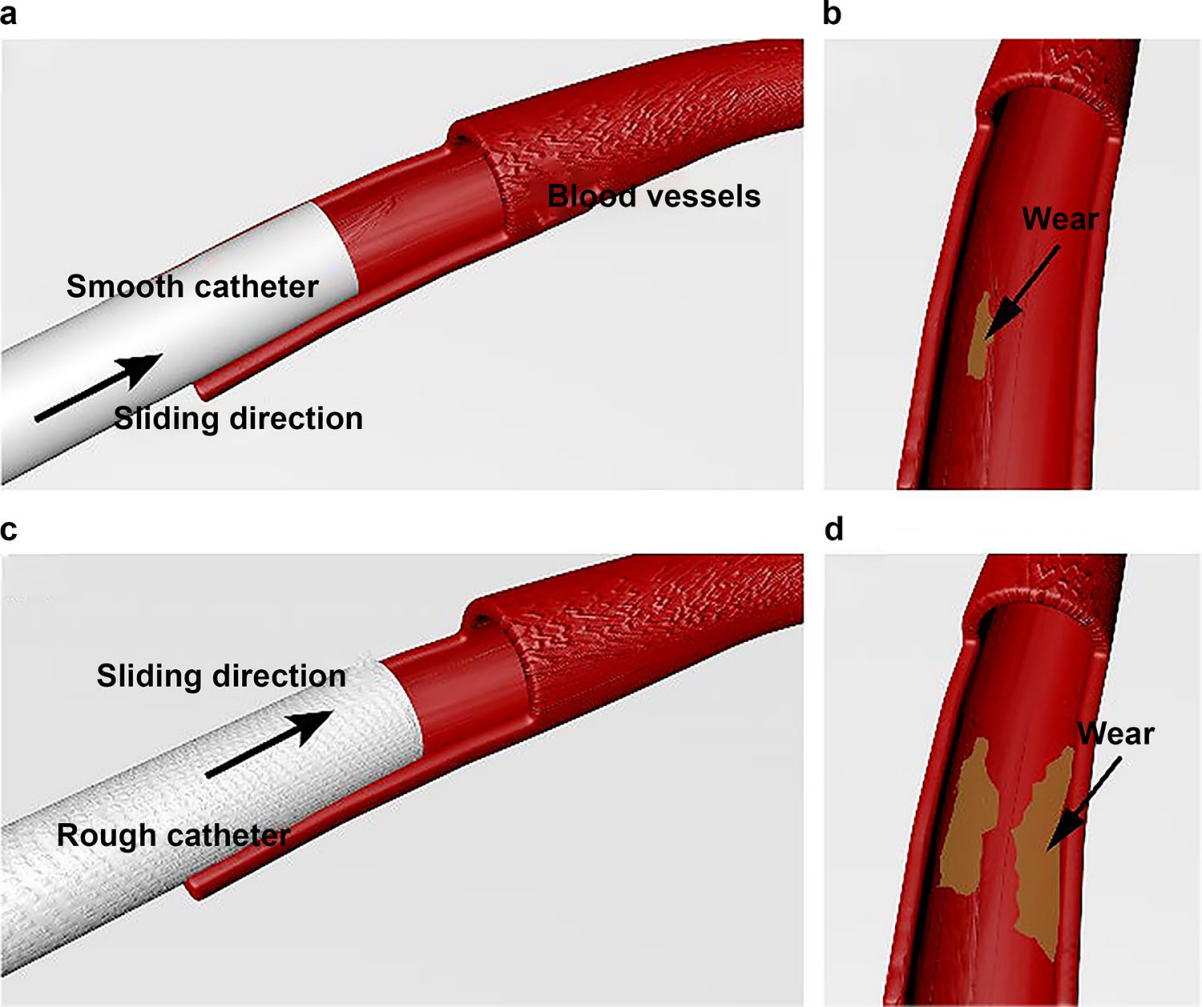
Simulation of the difference in friction damage caused by catheters of different roughness to the vascular intima.

The results of SEM and 3D white-light interferometry showed that the surface microstructure of the catheter was not smooth, particularly in some angles or sections, such as the tip of catheter where the roughness was relatively high. The morphology and roughness of the vascular intima also changed before and after the friction in vitro experiment, which can indicate that the catheter caused mechanical friction injury to the vascular intima during linear friction. Statistical analysis of the experiment confirmed that the surface roughness of the catheter positively correlated with the friction coefficient when it contacted the vascular intima, which was consistent with our initial hypothesis and results of various vascular injuries caused by indwelling catheters, as observed in previous studies. The catheter was originally designed to provide adequate blood flow while limiting recirculation rates^32^. Previous studies suggested that the macroscopic morphology of catheter tip may cause vascular damage^13^. Our study reveals, for the first time, that differences in the microscopic morphology of the roughness of catheters also have an impact on central venous endothelial damage. Therefore, when the catheter is placed in the central vein for a long time, the HD catheter with high surface roughness may cause relatively serious and continuous mechanical friction damage to the vascular intima (Fig. 6) and may be more likely to induce CVS and thrombosis.

At present, little attention has been paid to chronic mechanical friction trauma induced by catheters for HD on the central veins, including the superior vena cava, and no specific studies have been conducted. In addition, quantitative evaluation of the surface roughness of CVCs is lacking worldwide, and no clear standard and management of catheter surface roughness has been established. This study suggests that friction injury from rough catheter surfaces is a potential inducing factor for CVS and thromboembolism in patients with long-term indwelling CVC, which is worthy of attention. This result may serve as an entry point to reduce CVC-related complications and improve patient prognosis. In the future, more in vitro and clinical studies are needed to clarify the mechanism and downstream effects of friction damage between catheters and central veins, formulate clear parameters and management norms for catheter surface roughness, and promote the development of catheter surface lubrication technology. Essentially, not only CVCs are used for dialysis; peripherally inserted central catheters (PICCs) also have similar problems. They migrate with respiration, bloodstream flow dynamics, postural rotation and neck movements, injuring the blood vessel wall by rubbing^33^; thus, this experimental outcome may extend to PICCs.

## Limitations

Owing to the lack of previous research and data for reference, the parameters of this in vitro friction experiment were mainly set based on imaging findings and the clinical experience of experts, and referred to the initial preliminary experiment data. The parameters of in vitro experiments may differ from those in vivo, such as specific load force and friction velocity values between the catheter and the vascular intima. The simulated conditions in vitro cannot exactly replicate the biomechanical and hydrodynamic characteristics of the in vivo environment. However, as we are comparing the friction of different catheters with roughness on the vascular intima under the same experimental conditions, the conclusion remains of great reference value. The catheters used in this study did not cover all types of catheters, and the sample size was relatively small, so conclusions may not apply to all kinds of catheters. This study is the initial and basic stage of revealing the impact of microstructure and partial physic characteristics in catheter surfaces on mechanical friction injury to the intima. Further studies are needed to explore the biochemical reactions, inflammatory responses and coagulation mechanisms triggered by this, which are all critical factors in the development of catheter-related complications.

## Conclusions

In this study, we analyzed the microstructure of CVCs for HD and established an in vitro model to simulate the friction of the catheter on the vascular intima. The results revealed significant variations in the microscopic surface morphology of CVCs, which caused mechanical friction damage to vascular intima. To our knowledge, this is the first report on the impact of the microstructure and partial physic characteristics of catheter surface on the chronic injury of vascular intima. This study found an entry point for reducing CVC-related complications and improving patient prognosis, laid the foundation for subsequent in vivo studies to further validate the correlation between mechanical friction damage from CVCs and vascular injuries such as CVS, and provided a reference for guiding catheter production standards and management protocols, as well as promoting the development of catheter surface lubrication technology.

## Materials and methods

### Materials

The CVCs used in the experiment were as follows: Biometrix Hemo-Access 12-Fr 15-cm Double Lumen Dialysis Catheter (BM), ABLE Disposable Hemodialysis Access Catheter 11.5 Fr 20 cm (AB), COVIDIEN MAHURKAR Acute Dual Lumen Catheter 13.5 Fr 13.5 cm (CDA), COVIDIEN Permcath Chronic Silicone Oval Catheter 14.5-Fr 23-cm Dual Lumen Catheter (CDC), Medcomp 14.5Fr-24 cm PRE-CURVED Hemo-Flow Double Lumen Catheter (MC), and ARROW Cannon II Plus Hemodialysis Catheterization Set Long-Term Access 15 Fr 19 cm (AR). All brands of CVCs used in this study were made of polyurethane. The vessels for the experiments were taken from the superior and inferior vena cava of Japanese big-ear rabbits by Beijing Wanhe Technology Co., Ltd. (Beijing, China, Animal certificate no. 110329221100021361, Laboratory animal use certificate no. SYXK (京) 2020-0008). All vessels were preserved in 0.9% saline, and morphological observations and friction experiments were performed as soon as possible within 24 h. The study was approved by the Ethics Committee of the Emergency General Hospital (K22-7). All methods used followed relevant guidelines and regulations.

### Methods

#### Establishing an in vitro model and friction coefficient of the experimental catheter

Friction experiments were conducted in the SKLT Laboratory of Tsinghua University using a Universal Micro Tribometer (BRUKER UMT5) in the linear reciprocation mode. For the lower specimen, the central veins of the rabbit were attached to a 75-mm diameter Petri dish (intima upward). Approximately 30 mL of 0.9% saline was placed into the Petri dish to ensure that the samples were immersed completely. A special clamp was designed to fix the upper specimen—the CVCs should keep their major axis parallel to the long axis of the vascular sample during each trial. Based on the dynamic displacement of the catheter in the human central vein observed by angiography and other imaging methods, the loading force of the catheter body or tip in contact with the vessel was set to 0.5 N, stroke to 3 mm, and sliding speed to 3 mm/s. The friction coefficient was obtained by five measurements under the same conditions.

#### Scanning electron microscopy of the morphology of the vein intima after inducing friction

An environmental scanning electron microscope (SEM, Quanta200), which belongs to the Biomedical Test Center of Tsinghua University, was used to observe the morphology of catheters (tips and bodies) and the vascular intima (before and after friction).

#### 3D white-light interferometry for the quantitative assessment of the roughness of the testing catheters and intima

3D white-light interferometry (ZYGO Nexview, belonging to SKLT of Tsinghua University) was used to quantitatively assess different sites of the testing catheters (tips and bodies) and the vessel intima (before and after friction). For the sampling site, the catheter tip was selected, and annular sampling was performed randomly on the tube body 3 cm from the tip. The interval between sites was 120°. For the vessel intima, points were randomly taken along the central friction trajectory of the intima.

#### Statistical analysis

SPSS Statistics for Windows version 18.0 (SPSS Inc., Chicago, USA) was used to analyze the experimental data. Measurement data were expressed as mean ± standard deviation. One-way analysis of variance (ANOVA) was then used to assess whether significant differences existed between tips and bodies of different brands of catheters. Pearson's correlation analysis was used to evaluate the correlations of roughness and COF of the catheters. P < 0.05 was considered statistically significant.

### Ethics approval and consent to participate

This study was approved by the Emergency General Hospital Ethics Committee (Approval No. K22-7).

## Data Availability

The data presented in this study are available on reasonable request from the corresponding author.

## References

[1] Field M, Tullett K, Khawaja A, Jones R, Inston NG (2020). Quality improvement in vascular access: The role of patient-reported outcome measures. J. Vasc. Access.

[2] Lok CE (2020). KDOQI clinical practice guideline for vascular access: 2019 update. Am. J. Kidney Dis..

[3] Mengdi W (2016). Multi-center investigation of initial hemodialysis vascular access in end-stage renal disease patients. Chin. J. Nephrol..

[4] Htay H (2021). Hemodialysis use and practice patterns: An international survey study. Am. J. Kidney Dis..

[5] Hayes WN, Tennankore K, Battistella M, Chan CT (2014). Vascular access-related infection in nocturnal home hemodialysis. Hemodial. Int..

[6] Vô B, Anthonissen B, Verger C, Jadoul M, Morelle J, Goffin E (2022). Characteristics, practices, and outcomes in a Belgian cohort of incident home hemodialysis patients: A 6-year experience. Hemodial. Int..

[7] Allon M (2019). Vascular access for hemodialysis patients: New data should guide decision making. Clin. J. Am. Soc. Nephrol..

[8] Gan W (2023). The effect of early conversion from central venous catheter to arteriovenous fistula on hospitalization and mortality in incident haemodialysis patients. J. Vasc. Access.

[9] Masud AE, Costanzo EJ, Zuckerman R, Asif A (2018). The complications of vascular access in hemodialysis. Semin. Thromb. Hemost..

[10] Adwaney A, Lim C, Blakey S, Duncan N, Ashby DR (2019). Central venous stenosis, access outcome and survival in patients undergoing maintenance hemodialysis. Clin. J. Am. Soc. Nephrol..

[11] Tedla FM, Clerger G, Distant D, Salifu M (2018). Prevalence of central vein stenosis in patients referred tovein mapping. Clin. J. Am. Soc. Nephrol..

[12] Chan MR (2008). Hemodialysis central venous catheter dysfunction. Semin. Dial..

[13] Collier PE (2019). Prevention and treatment of dilator injuries during central venous catheter placement. J. Vasc. Surg. Venous Lymphat. Disord..

[14] Wang L, Jia L, Jiang A (2022). Pathology of catheter-related complications: What we need to know and what should be discovered. J. Int. Med. Res..

[15] de Oliveira DC (2021). Computational fluid dynamics of the right atrium: Assessment of modelling criteria for the evaluation of dialysis catheters. PLoS One.

[16] Garcia-Nicoletti M (2021). Silent and dangerous: Catheter-associated right atrial thrombus (CRAT) in children on chronic haemodialysis. Pediatr. Nephrol..

[17] Passaro ME, Steiger E, Curtas S, Seidner DL (1994). Long-term silastic catheters and chest pain. JPEN J. Parenter. Enteral Nutr..

[18] Agarwal AK (2009). Central vein stenosis: Current concepts. Adv. Chronic Kidney Dis..

[19] Miller LM (2016). Hemodialysis tunneled catheter noninfectious complications. Can. J. Kidney Health Dis..

[20] Sohail MA, Vachharajani TJ, Anvari E (2021). Central venous catheters for hemodialysis-the myth and the evidence. Kidney Int. Rep..

[21] Yevzlin AS (2008). Hemodialysis catheter-associated central venous stenosis. Semin. Dial..

[22] Al-Balas A, Almehmi A, Varma R, Al-Balas H, Allon MD (2021). novo central vein stenosis in hemodialysis patients following initial tunneled central vein catheter placement. Kidney.

[23] Forauer AR, Theoharis C (2003). Histologic changes in the human vein wall adjacent to indwelling central venous catheters. J. Vasc. Interv. Radiol..

[24] Kazmierska K, Szwast M, Ciach T (2008). Determination of urethral catheter surface lubricity. J. Mater. Sci. Mater. Med..

[25] Lin C, Huang Z, Wu T, Zhou X, Zhao R, Xu Z (2022). A chitosan and hyaluronic acid-modified layer-by-layer lubrication coating for cardiovascular catheter. Colloids Surf. B Biointerfaces.

[26] Wei Q, Liu X, Yue Q, Ma S, Zhou F (2019). Mussel-inspired one-step fabrication of ultralow-friction coatings on diverse biomaterial surfaces. Langmuir.

[27] Leblanc M, Bosc JY, Paganini EP, Canaud B (1997). Central venous dialysis catheter dysfunction. Adv. Ren. Replace. Ther..

[28] Curelaru I, Gustavsson B, Hansson AH, Linder LE, Stenqvist O, Wojciechowski J (1983). Material thrombogenicity in central venous catheterization II. A comparison between plain silicone elastomer, and plain polyethylene, long, antebrachial catheters. Acta Anaesthesiol. Scand..

[29] Sumiyoshi T (2015). A case of delayed vascular injury as a complication related to implanted central venous port catheter. Gan To Kagaku Ryoho.

[30] Sohail MA, Vachharajani TJ, Anvari E (2021). Central venous catheters for hemodialysis-the myth and the evidence. Kidney Int. Rep..

[31] Lin C, Kaper HJ, Li W, Splinter R, Sharma PK (2020). Role of endothelial glycocalyx in sliding friction at the catheter-blood vessel interface. Sci. Rep..

[32] Huriaux L, Costille P, Quintard H, Journois D, Kellum JA, Rimmelé T (2017). Haemodialysis catheters in the intensive care unit. Anaesth. Crit. Care Pain Med..

[33] Chica J, Ballén NP, Aguillon KJ, Rugeles SJ (2021). Hydromediastinum and hydrothorax as delayed complications of peripherally inserted central catheter used for Total Parenteral Nutrition: A case report. Int. J. Surg. Case Rep..

